# Dual Inhibition of SRC Family Kinases and Sorafenib Enhances Anti-Tumor Activity in Hepatocellular Carcinoma Cells

**DOI:** 10.3390/ijms26136506

**Published:** 2025-07-06

**Authors:** Loraine Kay Cabral, Cyrollah Disoma, Paola Tarchi, Korri Elvanita El-Khobar, Agustiningsih Agustiningsih, Francesco Dituri, Claudio Tiribelli, Caecilia Sukowati

**Affiliations:** 1 Liver Cancer Unit, Fondazione Italiana Fegato ONLUS, AREA Science Park, Campus Basovizza, 34149 Trieste, Italy; 2 Philippine Council for Health Research and Development, Department of Science and Technology, Saliksik Building, Science Community Complex General Santos Ave., Bicutan, Taguig City 1631, Philippines; 3 Doctoral School of Molecular Biomedicine, Department of Life Sciences, University of Trieste, 34149 Trieste, Italy; 4 General Surgery Department, Azienda Sanitaria Universitaria Giuliano Isontina, 34128 Trieste, Italy; 5 Eijkman Research Center for Molecular Biology, Research Organization for Health, National Research and Innovation Agency of Indonesia (BRIN), Jakarta Pusat 10340, Indonesia; 6 Personalized Medicine Laboratory, National Institute of Gastroenterology “S. De Bellis” IRCCS Research Hospital, Via Turi 27, 70013 Bari, Italy; francesco.dituri@irccsdebellis.it

**Keywords:** hepatocellular carcinoma, SRC family kinases, sorafenib, cellular heterogeneity

## Abstract

Hepatocellular carcinoma (HCC) remains a major clinical challenge due to its high recurrence rate and limited response to monotherapies, such as sorafenib—the standard first-line therapy for advanced HCC. This is partly attributed to its cellular heterogeneity. Increasing evidence implies SRC family kinase (SFK) activation in HCC progression, highlighting the potential of SRC-targeted therapies. In this study, we observed that *SRC* and *YES1* were significantly upregulated in clinical HCC specimens compared to its adjacent non-tumoral tissues (*p* < 0.001), suggesting relevance as therapeutic targets. High *SRC* expression was noticed in patients with poor prognosis, as confirmed in TCGA cohort. To evaluate the efficacy of dual targeting, we assessed the combination between SRC inhibitors, saracatinib and dasatinib, with sorafenib in six hepatic cell models, representing both S1 and S2 subtypes. Cytotoxicity assays demonstrated reduced cell viability with the combination therapies compared to either monotherapy, irrespective of the HCC subtype. Wound healing and Transwell migration assays revealed inhibition of cell migration and invasion following combination treatment, underscoring its potential to suppress metastatic behavior. RT-qPCR analysis further confirmed downregulation of the expression of *MMP2* and *MMP9*, genes associated with HCC cell invasion. Additionally, combined therapies decreased *VEGFA* and *HIF1A* expression compared to sorafenib alone, suggesting a potential to counteract the adaptive resistance mechanisms of cells to sorafenib. In summary, the combination of SFK inhibitors with sorafenib significantly enhances anti-tumor activity, offering a promising strategy to address HCC cellular heterogeneity and improve treatment efficacy.

## 1. Introduction

Despite the recent medical advances, the global burden due to hepatocellular carcinoma (HCC) continues to rise over the years. In 2022, HCC ranked sixth in terms of incidence with more than 860 thousand new cases and third in mortality with more than 750 thousand deaths [1,2]. HCC, a cancer of the hepatic parenchyma, presents in patients with chronic liver inflammation associated with viral infection, excess alcohol consumption, and metabolic dysfunction-associated steatotic liver disease. The poor prognosis is mainly attributed to several factors including late diagnosis, recurrence, metastasis, and drug resistance. For patients in late-advanced stages, only a handful of therapies can offer significant benefits, with available first-line systemic treatment of either tyrosine kinase inhibitors or immune-oncological approaches.

Together with the diverse etiological factors, hepatocarcinogenesis involves a muti-step process that constitutes the prolonged transformation of the cells, resulting in cellular inter- and intra-tumoral heterogeneity [3]. They have major implications for diagnosis and therapy and at the same time contribute largely to the chemoresistant nature of HCC, making the treatment more challenging. Several important studies were able to categorize the heterogeneous nature of HCC tumors by grouping them based on shared cellular and molecular characteristics. A pioneering study by Lee and Thorgeirsson was among the first to identify previously unrecognized, clinically relevant subclasses of HCC using gene expression profiling, leading to the stratification of phenotypic subtypes of HCC [4]. Building on this, Boyault et al. proposed a classification system comprising six subgroups (G1 to G6), each defined by distinct clinical and genetic profiles [5], while Hoshida et al. incorporated clinical parameters and identified three subclasses, including S1 (characterized by aberrant WNT signaling pathway activation), S2 (high EpCAM expression and activation of MYC and AKT pathways), and S3 (tumors with hepatocyte-like differentiation). Both classifications are correlated, both in clinical samples and diverse HCC cell lines [6,7]. These studies prompted the notion that in vitro HCC models could effectively replicate patient tumor characteristics and offer practical tools for biomarker discovery and drug response evaluation.

Sorafenib, a multi-target tyrosine kinase inhibitor, exhibits antiangiogenesis and antiproliferation effects and extends the total median survival in advanced HCC patients. Sorafenib suppresses tumor cell proliferation by inhibiting Raf-1, B-Raf, and kinase activity in the Ras/Raf/MEK/ERK signaling pathways [8]. It targets vascular endothelial growth factor receptor 2 (VEGFR2), platelet-derived growth factor receptor, hepatocyte factor receptor, fibroblast growth factor receptor, and other signaling targets, facilitating apoptosis, mitigating angiogenesis, and suppressing tumor cell proliferation. Unfortunately, about 30% of patients that benefit from sorafenib will eventually develop drug resistance within 6 months [9]. Resistance to sorafenib is attributed to several factors including genetics, epigenetics, transport process, regulated cell death, and tumor microenvironment [10].

Related with HCC cellular heterogeneity, previous studies have identified several therapy target molecules that might comprise HCC heterogeneity, particularly the Src family kinases (SFKs), a family of non-receptor tyrosine kinases (NRTKs). As NRTKs, SFKs bind to various proteins and act as regulators of the signal transduction pathways of many cellular processes [11]. There are nine members of SFKs, with SRC as the first identified member, and their dysregulations have been linked to various human cancers including HCC [11,12]. In this paper, we demonstrated that the cytotoxicity effect of sorafenib is improved in combination with SFK inhibition in in vitro HCC cell models.

## 2. Results

### 2.1. Identification of SFKs as Targets for Therapy

In our previous study, utilizing comprehensive information on HCC-omics heterogeneity, we implemented a strategy to identify putative markers for HCC treatment. We focused on cancer-promoting genes commonly shared across the defined subclasses and subgroups and evaluated 16 potential targets at the transcriptome level in response to three different treatment modalities in five HCC cell lines. HCC cells HLE, HLF, and JHH6 were classified to subtype 1/transforming growth factor beta-Wingless related integration site (S1/TGFβ-Wnt) activated subtype, while Huh7 and HepG2 were classified as subtype 2 (S2/progenitor subtype) [6,13].

The set of 16 proto-oncogenes for HCC that targeted the molecules above [13] was then analyzed using bioinformatic tools to understand the functions and interaction. STRING analysis [14] revealed a significant protein–protein interaction (PPI) enrichment (*p*-value = 4.17 × 10^−10^), suggesting that the 16 proteins are at least partially biologically connected as a group. Gene enrichment analysis using g.profiler [15] further highlighted a distinct subset of non-membrane tyrosine kinases belonging to the SFKs. Notably, four of the nine SFK members—*SRC*, *FGR*, *YES1*, and *FYN*—were among the predicted targets, drawing our attention to this unique clustering within the protein interaction network. Supporting data are presented in Supplemental Figure S1.

### 2.2. SRC as Prognostic Tool in HCC Patients

**Figure 1:**
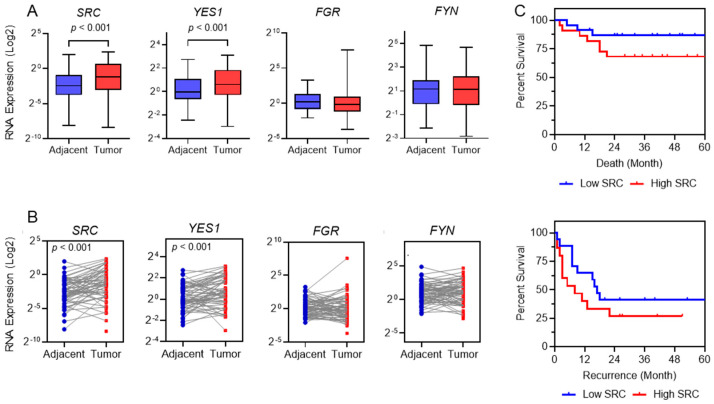
Src family kinases family gene expression in human HCC specimens (*n* = 61 tissue pairs). (**A**,**B**) Significant increase in *SRC* and *YES1* expression in tumors compared to their paired adjacent non-tumoral tissues (paired Student’s *t*-test *p* < 0.001). (**C**) High *SRC* expression in HCC nodules (defined as median mRNA level cut-off value of 0.50 a.u) is noticed in patients with poorer survival within 24 months. Graph is presented until 60 months of follow-up. All gene expression data were normalized to reference genes *GAPDH* and *18S-rRNA*.

To investigate SFKs’ potential role in HCC development, we analyzed the gene expression levels of four SFK members—*SRC*/*ASV1*, *FGR*, *YES1*, and *FYN*—in human clinical specimens using reverse transcription-quantitative real time polymerase chain reaction (RT-qPCR). Paired tissue samples, consisting of tumor (HCC nodule) and adjacent non-tumoral liver tissues, were obtained from 61 HCC patients (mean age: 54 years; 48 males, 13 females). As shown in Figure 1A,B, *SRC* expression was significantly upregulated in tumor tissues compared to matched non-tumorous tissues (paired Student’s *t*-test, *p* < 0.001). A similar trend was observed for *YES1* (*p* < 0.001), whereas *FGR* and *FYN* did not show significant differences.

Regarding clinical outcomes, elevated *SRC* expression—defined as median cut-off value of 0.50 a.u. in HCC nodules—was noticed in patients with poorer prognosis after 24 months, both in terms of recurrence and overall survival, although the differences did not reach statistical significance (Figure 1C).

To validate these findings in a larger dataset, we assessed the differential expression of *SRC*, *FGR*, *YES1*, and *FYN* using the data from LIHC (liver hepatocellular carcinoma) of the Cancer Genome Atlas (TCGA) and the Genotype Tissue Expression (GTEx) portals [16,17]. We analyzed and visualized the data using the Gene Expression Profiling Interactive Analysis (GEPIA) online tool [18]. The prognostic relevance of SFK gene expression on overall survival was evaluated using validated data of The Human Protein Atlas (HPA) that uses the TCGA LIHC cohort [19].

In the TCGA LIHC and GTEx datasets, *SRC* and *YES1* were also significantly upregulated in tumor tissues relative to normal liver tissues (*p* < 0.01 for both), while *FGR* and *FYN* remained unchanged (Supplemental Figure S2A). Prognostic analysis using the HPA further demonstrated that high expression of *SRC* and *YES1* was significantly associated with reduced overall survival in LIHC patients (*p* < 0.01 and *p* < 0.005, respectively) (Supplemental Figure S2B).

### 2.3. Combination Therapy Increases Sorafenib Cytotoxicity in HCC Cell Lines

Given that both clinical samples and TCGA analysis revealed significant differential expression of *SRC* and *YES1* across samples, we investigated whether pharmacological inhibition of SFKs could potentiate the therapeutic efficacy of sorafenib. The cytotoxic effects of two SFK inhibitors—saracatinib and dasatinib—were assessed using the MTT assay in different cell lines, including HCC subtype S1/TGFβ-Wnt (HLE, HLF, and JHH6), HCC subtype S2/progenitor subtype (Huh7 and HepG2), and immortalized hepatocytes (IHHs) [6].

**Figure 2:**
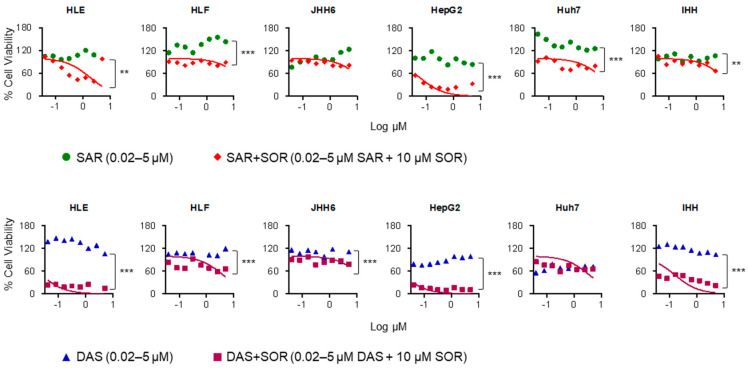
Cytotoxicity assay results of two Src family kinase inhibitors, saracatinib and dasatinib, alone and in combination with 10 µM sorafenib in different hepatic cell lines. All cells were treated with either saracatinib or dasatinib with a concentration ranging from 0.02 to 5.0 µM for 24 h. Two-tailed paired Student’s *t*-test, ** *p* < 0.01, *** *p* < 0.001. DAS: dasatinib; SAR: saracatinib, SOR: sorafenib.

Following 24 h treatment, neither saracatinib nor dasatinib exhibited significant cytotoxicity across all cell lines tested, where all treatments did not reach the LC_50_ (Figure 2). In parallel, the treatment with 10 µM sorafenib alone led to a moderate reduction in cell viability (70–90%) in HepG2, HLE, and IHH cells, while no appreciable cytotoxic effect was observed in HLF and JHH6 cells [13]. This dose was then selected to appreciate the cytotoxicity effects of combined therapies with saracatinib and dasatinib.

Using the 3(4,5-dimethyl thiazolyl-2)-2,5 diphenyltetrazolium (MTT) assay as a proxy for cell viability and the same concentration as monotherapy, the combined therapies of sorafenib with either saracatinib or dasatinib elicited a dose-dependent cytotoxic response. Among the tested cell lines, HepG2 and HLE demonstrated the highest sensitivity to co-treatment, exhibiting LC_50_ values of 0.06 µM and 0.6 µM for the saracatinib- sorafenib combination and 0.01 µM and 0.02 µM for the dasatinib-sorafenib combination, respectively. Meanwhile, HLF, JHH6, and Huh7 cells displayed relatively higher LC_50_ values of 5.7, 4.5, and 2.6 µM for the saracatinib-sorafenib combination and 1.8, 4.6, and 1.2 µM for the dasatinib-sorafenib combination, respectively. Importantly, in the non-tumorigenic IHH cell line, the saracatinib-sorafenib combination exhibited markedly lower cytotoxicity compared to the dasatinib-sorafenib combination, with LC_50_ values of 4.8 µM and 0.1 µM, respectively (Figure 2). The combination therapies showed significant differences in all cell lines (*p* < 0.05), except in Huh7 cells treated with dasatinib-sorafenib and JHH6 treated with saracatinib-sorafenib.

### 2.4. Combination Therapy Reduces Cell Aggressiveness and Migration Capacity

**Figure 3:**
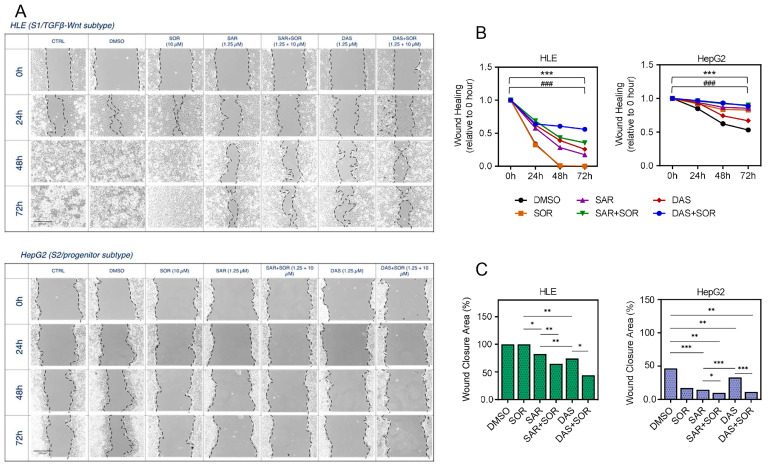
Wound healing assay. (**A**) Representative light microscope images of HLE (S1/TGFβ-Wnt subtype) and HepG2 (S2/progenitor subtype) at 0, 24, 48, and 72 hrs after treatment with saracatinib and dasatinib alone or in combination with sorafenib. Original objective magnification = 10×. (**B**) Wound healing rate of HLE and HepG2 cells after treatment, measured relative to 0 hr. Two-way ANOVA, *** *p* < 0.001 to drug exposure, ### *p* < 0.001 to drug combinations. (**C**) Percentage of the wound closure area in HLE and HepG2 cells. * *p* < 0.05, ** *p* < 0.01, and *** *p* < 0.001. CTRL: untreated control, DAS: dasatinib-treated cells, DMSO: DMSO-treated control cells, SAR: saracatinib-treated cells, SOR: sorafenib-treated cells.

We further evaluated the anti-migration effect of the SFK inhibitors as mono and combination therapy by analyzing the areas of wound healing capacity using scratch assay. Wound closure area was measured as the remaining area uncovered by the cells at 0, 24, 48, and 72 hrs (Figure 3A–C). HLE and HepG2 were selected to represent S1/TGFβ-Wnt and S2/progenitor subtype cells, respectively. Based on the wound area shown in Figure 3A,B, combined therapies of saracatinib-sorafenib or dasatinib-sorafenib resulted in a wider wound area compared to each of the single treatments, indicating for better cell migration inhibition ability of the combined therapies. Statistical analysis showed that drug combinations resulted in significant differences (*p* < 0.05) compared with DMSO-treated cells. Additionally, Figure 3C clearly showed that all treatments reduced the capacity of the cells to invade the wound space, as shown by the reduced percentages of the wound closure area, which were particularly noticed for the combinations of saracatinib-sorafenib and dasatinib-sorafenib (*p* < 0.05 for both combined therapies, in both cell lines).

**Figure 4:**
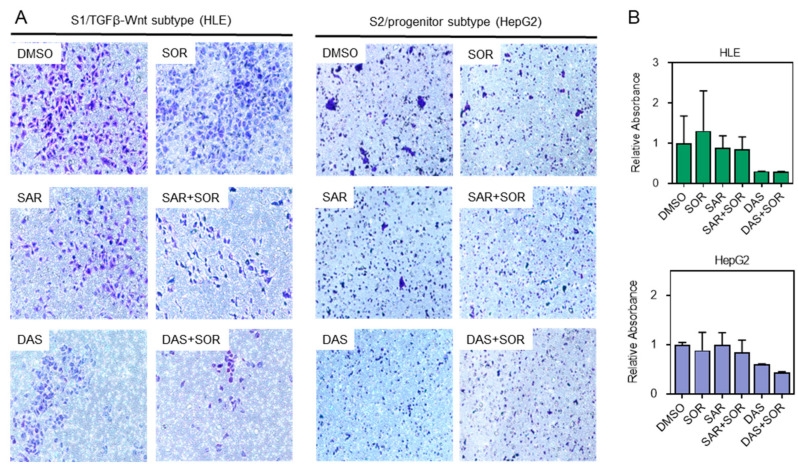
Transwell cell migration assay. (**A**) Representative light microscope images of HLE (S1/TGFβ-Wnt subtype) and HepG2 (S2/progenitor subtype) after treatment with saracatinib and dasatinib alone or in combination with sorafenib. Original objective magnification = 10×. (**B**) Relative absorbance of HLE and HepG2 after treatment. Data are presented as mean ± SEM from at least three independent replicates. DAS: dasatinib-treated cells, DMSO: DMSO-treated control cells, SAR: saracatinib-treated cells, SOR: sorafenib-treated cells.

In addition to the wound healing assay, we performed a Transwell cell migration assay to further validate the impact of the combination treatments on the migratory capacity of HCC cells. As shown in Figure 4A,B, both combination treatments—saracatinib-sorafenib and dasatinib-sorafenib—reduced the number of migrated cells compared to either treatment alone. This observation indicates that the combined therapies are more effective than monotherapies in impairing the migratory and invasive behavior of HCC cells.

### 2.5. Combination Therapy Dysregulated Gene Expression Related to Cell Migration and Angiogenesis

Following the functional assay, we focused next on the angiogenesis pathway, which is related to cancer cell aggressiveness. We performed gene expression analysis using RT-qPCR of *SRC* and *YES1* expression together with common targets of both SFK inhibitors and sorafenib to identify the molecular basis of the combined therapy. For this analysis, we selected HepG2 cells, which were highly sensitive to the combination treatment, and HLF cells, which are less sensitive (Figure 2). As noted above, these cell lines represent S2 and S1 cellular subtypes, respectively, and their use in functional assays aims to reflect the potential efficacy of the combined treatment across distinct cellular populations.

As expected, saracatinib or dasatinib alone reduced the expression of *SRC* in HepG2 and HLF by around 50% and 30%, respectively (*p* < 0.05 for HepG2), and *YES1* in HepG2 cells around 70% (*p* < 0.05). Interestingly, sorafenib was also observed to downregulate the expression of *SRC* in HLF. Regarding the combined therapies, however, the presence of sorafenib resulted in increased expression of *SRC* and *YES1* compared to SFK inhibitors alone, although the upregulation was not significant.

**Figure 5:**
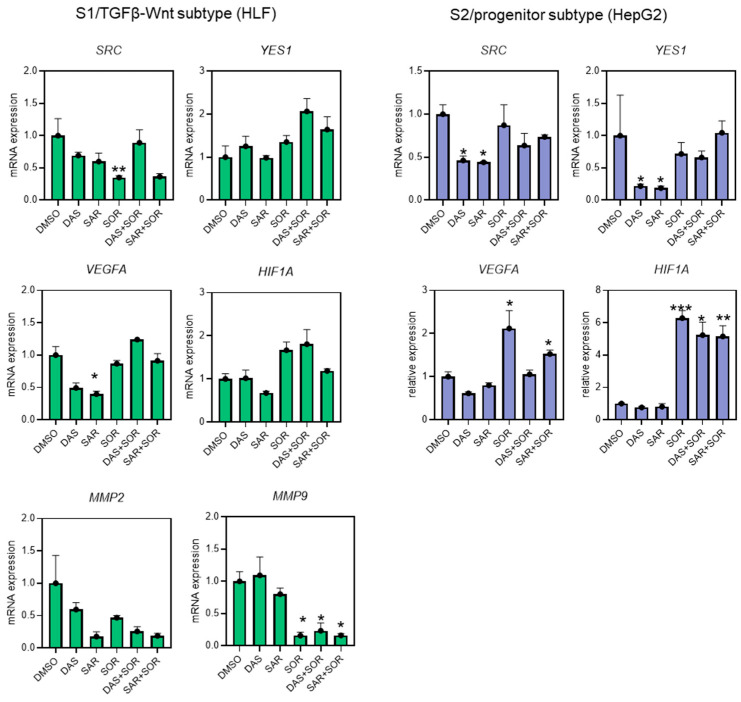
Relative gene expression of *SRC*, *YES1*, *VEGFA*, *HIF1A*, *MMP2*, and *MMP9* in the human HCC cell lines HLF (S1/TGFβ-Wnt subtype) and HepG2 (S2/progenitor subtype). DMSO control cells were set at 1.0. * *p* < 0.05, ** *p* < 0.01, and *** *p* < 0.001 vs. DMSO. All gene expression data were normalized to reference genes *GAPDH* and *18S-rRNA*. Data are presented as mean ± SEM from three independent replicates. DAS: dasatinib-treated cells, DMSO: DMSO-treated control cells, SAR: saracatinib-treated cells, SOR: sorafenib-treated cells.

Further analysis showed that the combination therapy in HLF cells significantly reduced the expression levels of matrix metallopeptidase 2 (*MMP2*) and matrix metallopeptidase 9 (*MMP9*), two key matrix metalloproteinases associated with extracellular matrix degradation and the invasive potential of HCC cells, thus correlating the molecular basis of the functional assays. In addition to its anti-invasive effects, the combination therapy also led to a marked decrease in the expression of vascular endothelial growth factor A (*VEGFA*) and hypoxia-inducible factor 1-alpha (*HIF1A*), both regulators of angiogenesis, compared to sorafenib monotherapy, thereby enhancing treatment efficacy and potentially overcoming resistance to sorafenib (Figure 5).

## 3. Discussion

Combination therapy, which involves using two or more agents to target cancer-promoting or sustaining pathways, is a well-established strategy in oncology [20,21]. While monotherapy remains common for many cancers, it often proves less effective than combination approaches. However, increased therapeutic efficacy with combination regimens can sometimes be accompanied by greater toxicity. Notably, multi-targeted therapies may allow for reduced dosages of individual agents, thereby minimizing off-target or cytotoxic effects on healthy tissues [22,23].

Following our previous study where we implemented an in silico strategy to identify putative targets for HCC treatment that may comprise HCC vast cellular heterogeneity [13], we further identified SFKs as potential therapeutic targets in HCC. SFKs are involved in multiple signaling pathways associated with malignant transformation and tumor progression [24]. This family comprises nine structurally similar non-receptor tyrosine kinases, including SRC, FYN, YES, LYN, BLK, FGR, HCK, YRK, and LCK [25], with SRC, FYN, YES, and FGR forming the closely related group I enzymes. Although SRC itself is not typically an initiating factor in tumorigenesis, it plays a pivotal role in supporting tumor growth and survival. It acts downstream of oncogenic drivers such as EGFR, ErbB2, and BCR-Abl and is involved in major pathways like Ras/Raf/ERK and PI3K/Akt, which regulate cell proliferation and survival, respectively [22].

To assess the role of SFKs in hepatocarcinogenesis, we analyzed their gene expression in a cohort of HCC patients who underwent liver resection without prior treatment. We found that *SRC* and *YES1* were significantly upregulated in tumor tissues compared to adjacent non-tumoral liver tissues (*p* < 0.001, Figure 1), confirming their relevance as therapeutic targets in this study. Larger data from TCGA cohort confirmed our data, as also shown by its significance in the prognosis of HCC patients. These findings are consistent with previous reports showing increased expression and activation of these kinases in HCC tissues [23,26,27]. Notably, SRC has been shown to promote HCC cell growth and tumorigenesis through activation of the Hippo signaling pathway [28].

To explore the anti-tumor potential of SFK inhibition, we tested the effects of saracatinib and dasatinib, both alone and in combination with sorafenib, a multikinase inhibitor, in a panel of in vitro HCC models representing both S1 and S2 subtypes. These models, which recapitulate distinct key molecular features of patient tumors, serve as practical tools for evaluating drug responses and biomarker discovery [6,13]. The combination of SFK inhibitors with sorafenib represents a dual-targeting approach: receptor tyrosine kinases (RTKs) via sorafenib and NRTKs via SFK inhibitors. A recent study has shown that treatment with sorafenib or dasatinib markedly inhibited the growth of HepYF, an aggressive proliferation class G3 subgroup of HCC, in an animal model [29]. To our knowledge, this is the first preclinical study investigating the combination of two SFK inhibitors, saracatinib and dasatinib, and sorafenib in HCC in relation to various HCC cellular types.

Dasatinib is the only FDA-approved Src-Abl inhibitor, indicated for chronic myelogenous leukemia and Philadelphia chromosome-positive acute lymphocytic leukemia in patients who have failed first-line therapy [30,31]. It has also shown anti-tumor activity in various solid tumors, prompting multiple phase I and II trials, both as monotherapy and in combination therapies [32].

Saracatinib is another orally available, selective Src-Abl inhibitor with demonstrated preclinical efficacy across several solid tumor models [33,34,35]. Phase I trial data for advanced solid tumors identified the maximum tolerated dose as 175 mg daily (versus 120 mg for dasatinib), but saracatinib’s side effects were more manageable despite the higher dose [36]. Both drugs exhibit distinct inhibitory profiles: dasatinib shows moderate activity against Lck, Fyn, and Yes1 [37], while saracatinib strongly inhibits Src1, Yes1, Lck, and Abl [35].

In our cytotoxicity assays, monotherapy with sorafenib (10 µM), saracatinib or dasatinib (0.02–5.00 µM) resulted in minimal toxicity across diverse HCC cell lines and immortalized hepatocytes (IHHs) (Figure 2). However, when saracatinib or dasatinib was combined with sorafenib, a substantial reduction in cell viability was observed. Interestingly, the saracatinib-sorafenib combination was less toxic to IHHs than dasatinib-sorafenib at equivalent concentrations, suggesting a better safety profile. These results align with clinical observations of saracatinib’s manageable toxicity in solid tumor trials [36,38]. However, since the concentrations of drugs used in the study showed minimal to moderate reduction in cell viability when each drug was used individually—that is, below the calculated LC_50_—the combination index [39] for drugs combination was not calculated in this study.

Functional validation using scratch wound healing and Transwell migration assays showed that combination therapies significantly impaired cell migration and invasion in HLE (S1 subtype) and HepG2 (S2 subtype) cells compared to monotherapies. Wound closure was notably inhibited as early as 24 h after treatment and remained so at 72 h (Figure 3A–C). Similar findings were seen in migration assays, where fewer cells traversed the membrane after combined treatment (Figure 4A,B).

To investigate the molecular mechanisms underlying these effects, we conducted RT-qPCR analysis in HepG2 and HLF cells to examine changes in gene expression. HepG2 cells, which exhibit high sensitivity to the combination treatment, and HLF cells, which show lower sensitivity, were selected to represent the S2 and S1 cell types, respectively. This approach allows us to assess the potential efficacy of the combined treatment across distinct cellular subpopulations based on their differential responsiveness. Although functional assays were performed in HepG2 and HLE cells, HLF was included in the molecular analysis to provide a more accurate representation of less responsive cell types. Importantly, HLE and HLF cells originate from the same HCC patient [40], offering a relevant model to study intra-tumoral heterogeneity and the variable response to combination therapy within a single individual.

The combination therapies significantly downregulated *MMP2* and *MMP9*, which are key to extracellular matrix remodeling and invasion [41] (Figure 5). Additionally, expression of *VEGFA* and *HIF1A*, crucial for angiogenesis and hypoxia adaptation [42], were reduced in combination therapy compared to sorafenib alone. This suggests that SFK inhibition may enhance sorafenib’s anti-angiogenic effects and counteract resistance mechanisms.

Sorafenib and SRC inhibitors target distinct yet intersecting oncogenic pathways in HCC, which likely underlies their observed synergistic effects. Both drug classes converge on critical signaling cascades, including the MAPK/ERK and STAT3 pathways, which are pivotal for tumor cell proliferation, survival, and migration [43,44,45]. In addition, SRC plays a key role in mediating VEGFR signaling and contributes to angiogenesis and vascular permeability, overlapping mechanistically with the anti-angiogenic properties of sorafenib [46,47]. SRC inhibitors further interfere with the SRC–focal adhesion kinase (FAK) complex, which is essential for cellular adhesion and migration [48], whereas sorafenib has also been reported to impact focal adhesion signaling [49].

A previous study using a lung cancer xenograft model demonstrated that combination treatment with sorafenib and dasatinib produced a strong anti-proliferative effect in A549 human lung adenocarcinoma cells. The study further identified FAK/SRC activation as a driver of acquired resistance to sorafenib by promoting epithelial–mesenchymal transition and invasive behavior [50]. Notably, SRC inhibition was insufficient to fully overcome sorafenib resistance, which was consistent with our findings. While the addition of saracatinib or dasatinib significantly downregulated *VEGFA* and *HIF1A* expression, the reduction did not reach basal levels observed in untreated controls (Figure 5). These findings support the rationale for combination therapy targeting both SRC and RAF/VEGFR pathways in HCC.

Despite the overall efficacy, response levels varied among different HCC cell lines, reflecting tumor heterogeneity. Future studies should aim to identify molecular biomarkers predictive of sensitivity to such combination therapies. This is consistent with findings from clinical trials in other cancers. For example, a Phase II study showed that saracatinib may benefit a subpopulation of non-small cell lung cancer (NSCLC) patients with epidermal growth factor receptor mutations [38]. Current clinical trials are exploring biomarkers to guide SRC inhibitor therapies and identify patients most likely to benefit [34].

In summary, our data demonstrates that dasatinib and saracatinib, when combined with sorafenib, significantly enhance anti-tumor effects in HCC cell lines at tolerable doses. This combination therapy shows promise in overcoming the limitations of monotherapy and addressing the heterogeneity inherent in HCC tumors.

## 4. Materials and Methods

### 4.1. Bioinformatics Analysis

The set of 16 proto-oncogenes for HCC targeted molecules in our previous work [13] was analyzed to understand the functions, roles, and their association using bioinformatic tools using the STRING database [14]. Meanwhile, g.profiler tool [15] was used to identify gene enrichment analysis of the protein targets.

### 4.2. Human HCC Specimens

Sixty-one cases of HCC patients undergoing surgical resection without any prior treatments were included in this study. From each patient, paired liver tissues, one from the nodule (tumor; HCC) and one from its adjacent (non-tumor) tissues, were collected. Informed consent to participate in the study was obtained from each patient or by a legal representative, and patient anonymity has been preserved. Investigation was conducted according to the principles expressed in the Declaration of Helsinki. The human samples collection was approved by the regional ethical committee (Comitato Etico Regionale Unico del Friuli Venezia Giulia no. 1554). Immediately after surgery, fresh liver tissues were collected and snap-frozen in liquid nitrogen and stored at −80 °C.

### 4.3. SFK Genes Expression and Prognostic Significance

The distributions of SFK family members belonging to the target genes were assessed in human clinical specimens, by comparing their expression in nodules compared to its adjacent non-tumoral tissues. Survival was analyzed using Kaplan–Meier curves and the log-rank test. Patients were classified into two groups (high and low expression) based on the best expression cut-off yields maximal difference with regards to survival at the lowest log-rank *p*-value.

The data from the LIHC TCGA cohort (https://portal.gdc.cancer.gov/) and the GTEx (https://www.gtexportal.org/home/) portals [16,17] were also analyzed and visualized using the Gene GEPIA online tool, accessed on 1 February 2025 [18]. The prognostic relevance of SFK gene expression on overall survival was evaluated using validated data of The Human Protein Atlas (https://www.proteinatlas.org/, accessed on 1 February 2025) using the TCGA LIHC cohort [19].

### 4.4. Cell Lines

Representative cell lines that correspond to the different subtypes of HCC, which consisted of immortalized hepatocytes (IHHs) and five HCC cell lines, were used for in vitro analysis. HCC cell lines HLE, HLF, and JHH6 were classified under the subtype 1/transforming growth factor beta-Wingless related integration site (S1/TGFβ-Wnt) activated subtype, and HepG2 and Huh7 were classified as subtype 2 (S2/progenitor subtype) [6]. All cell lines were grown in their respective culture media supplemented with 10% (*v*/*v*) fetal bovine serum, 1% L-glutamine, and 1% antibiotics. Dulbecco’s modified Eagle’s medium (DMEM)-F12 medium was used for IHHs with additional supplements of 1 μM dexamethasone and 5 μg/mL insulin. DMEM medium (high glucose) was used for all HCC cells, except for JHH6 which was cultured in Williams’ E medium. Cells were maintained at 37 °C in a humidified 5% CO_2_ incubator. Routine cell expansion was performed using 0.05% trypsin detachment when cells achieved 80% cell confluency. Human HCC cell lines Huh7 (JCRB0403) and JHH6 (JCRB1030) were obtained from the Japan Health Science Research Resources Bank (HSRRB, Tokyo, Japan). The HepG2 cell line was obtained from the Istituto Zooprofilattico Sperimentale della Lombardia e dell’Emilia Romagna (IZSLER, Brescia, Italy). HLE and HLF cell lines were kindly provided by the laboratory of Dr. Giannelli of the National Institute of Gastroenterology S. De Bellis Research Hospital, Bari, Italy. IHH cells were kindly provided by Dr. Trono (Lausanne, Switzerland).

### 4.5. Cytotoxicity of Combination Therapy

For the evaluation of cytotoxicity of SFK inhibitors alone, including saracatinib (HY-10234) and dasatinib (HY-10181) (MedChemExpress, South Brunswick, NJ, USA), and their combination with sorafenib, all cells were treated with a concentration ranging from 0.02 to 5.0 µM for 24 h. In combined therapies, the sorafenib concentration was set to 10 µM to obtain an acceptable minimal to moderate reduction in cell viability for all cells analyzed [13]. As a control, the DMSO concentration was set to 0.1% in each treatment. After 24 h of treatment, cellular viability was evaluated using the MTT (Sigma-Aldrich, St Louis, MO, USA) as a proxy, to determine the LC_50_ of the drug to each cell line. The absorbance of calorimetric intensity was read using a plate multireader (Enspire, Perkin Elmer, Shelton, CT, USA).

### 4.6. Total RNA Extraction from Solid Tissue Samples and Cell Lines

Total RNA was isolated from fresh clinical tissue specimens stored in −80 °C freezer immediately after liver resection and from HepG2 and HLF cell lines following 24 h treatment with 5 µM of sorafenib and 1.25 µM of saracatinib and dasatinib. Total RNA was extracted using Tri Reagent^®^ (Sigma-Aldrich) according to the manufacturer’s protocol. Tissue homogenization was achieved using tissue grinders and cell scraping for solid tissue and cell lines, respectively. All homogenates were suspended in 500–1000 µL of Tri Reagent^®^, followed by phase separation, RNA precipitation, washing, drying, and RNA pellet dissolution. RNA was quantified at wavelength 260 nm in a spectrophotometer (Beckman Coulter, Brea, CA, USA), and RNA purity was evaluated according to the Minimum Information for Publication of Quantitative Real-Time PCR Experiments (MIQE) guidelines by measuring the ratio A260/A280 with an appropriate purity value between 1.8 and 2.0 [51]. The integrity of RNA was assessed using a standard 1% agarose/formaldehyde gel.

### 4.7. RT-qPCR

Reverse transcription was performed to obtain cDNA from 1 μg of purified RNA with the High-Capacity cDNA Reverse Transcription Kits (Applied Biosystems, Waltham, MA, USA) according to the manufacturer’s protocol. RT-qPCR was performed according to the SYBR Green Supermix protocol (Bio-Rad Laboratories, Hercules, CA, USA). PCR amplification was carried out in a 15 μL reaction volume containing 25 ng cDNA, 1 × iQ5 SYBR Green Supermix, and 100–250 nM of gene-specific forward and reverse primers. The reaction was run in CFX 9600 real-time PCR system (Bio-Rad). The primer sequences are designed using Beacon Designer 7.9 Software (PREMIER Biosoft International, Palo Alto, CA, USA) for the detection of the desired gene and are listed in Table 1.

### 4.8. Wound Healing Assay

HLE and HepG2 cells, representing S1 and S2 HCC subtypes, respectively, were grown in a 12-well plate with an initial concentration of 37,500 cells/cm^2^. After 24 h, a longitudinal scratch was introduced to the monolayer of cells in each well using the end of a white pipette tip, and the medium was replaced with new medium containing treatment set-ups. The concentration of drugs used were 10 μM of sorafenib, 1.25 μM of saracatinib, 1.25 μM of dasatinib, and their combinations. The migration capacity of the cells was recorded by taking representative photos of each treatment set-up in 6 different wound spots using an optical microscope (Leica, Solms, Hesse, Germany). The quantification was performed by measuring the wound area using ImageJ 1.54g (National Institutes of Health, Bethesda, MD, USA). Data were obtained at 0, 24, 48, and 72 h. Data were generated as the area of the percentage (%) of wound closure computed as follows: ((t0−th)/t0)) × 100%, where t0 is the area of the wound measured immediately after scratching, and th is the area of the wound measured “h” hours after the scratch is performed. Results are presented as means of the measurements of areas and standard deviation between the representative points.

### 4.9. Transwell Migration Assay

On a 24-well plate, the upper chamber of the Transwell inserts (Corning, New York, NY, USA) was seeded with 250 μL of cells in medium with treatment set-ups with 2% FBS with same concentration as above, while the lower chamber was filled with 500 μL of medium supplemented with 20% FBS. The concentration of drugs used were 10 μM of sorafenib, 1.25 μM of saracatinib, 1.25 μM of dasatinib, and their combinations. After 24 h, the upper chamber cells were removed, and the lower chamber cells were fixed with 4% paraformaldehyde, stained with crystal violet solution, observed, and recorded under an optical microscope (Leica). Quantitative analysis of migrating cells was performed using ImageJ software (National Institutes of Health). For absorbance measurements, crystal violet was dissolved in 300 µL of 1% SDS in a rotating platform for 1 h. Absorbance was read using a plate multireader (Enspire, Perkin Elmer).

### 4.10. Statistical Analysis

Statistical significance was calculated using GraphPad Prism version 8.0 (GraphPad Software, Inc., La Jolla, CA, USA). In vitro data were obtained from at least three independent experiments and are expressed as mean ± SD/SEM. Graphics, survival curves, and statistical analyses were constructed using GraphPad Prism. For the cytotoxicity assay, two-tailed paired *t*-test was used, whereas two-way ANOVA was performed for the wound-healing rate to determine the effect of drug exposure and drug combinations. Statistical significance was set to *p*-value < 0.05 and reported as indicated here: * *p* < 0.05, ** *p* < 0.01, and *** *p* < 0.001.

## Figures and Tables

**Table 1:** Primer sets used to assess gene expression in HCC cell lines.

Gene	Primer F (5′→3′)	Primer R (5′→3′)
*18SRNA*	TAACCCGTTGAACCCCATT	CCATCCAATCGGTAGTAGCG
*GAPDH*	CCCATGTTCGTCATGGGTGT	TGGTCATGAGTCCTTCCACGATA
*SRC/ASV1*	CGCTGGCCGGTGGAGTG	CCAGCTTGCGGATCTTGTAGT
*FGR*	GGCCCGGCCTGCAT	TTGATGGCCTGAGAGGAGAAG
*YES1*	ACAGCAAGACAAGGTGCAAA	GTAAACCGACCATACAGTGCAG
*FYN*	GGACATGGCAGCACAGGTG	TTTGCTGATCGCAGATCTCTATG
*HIF1A*	CCAGCAGACTCAAATACAAGAACC	TGTATGTGGGTAGGAGATGGAGAT
*VEGFA*	GTGAGGCGGCGGTGTG	GCAAGGCAAGGCTCCAATG
*MMP2*	CCAAGAATAGATGCTGACTG	GGAGAAGAGCCTGAAGTG
*MMP9*	CGGCAAGTCTTCCGAGTAGT	AGACCTGGGCAGATTCCAAAC

## Data Availability

The data that support the findings of this study are available on reasonable request from the corresponding author.

## Supplementary Materials

The following supporting information can be downloaded at: https://www.mdpi.com/article/10.3390/ijms26136506/s1. Reference [52] is cited in the supplementary materials.

## Abbreviations

HCC	Hepatocellular Carcinoma
SFKs	Src Family Kinases
NRTKs	Non-Receptor Tyrosine Kinases
RT-qPCR	Reverse Transcription-Quantitative Real-Time Polymerase Chain Reaction
RT	Reverse Transcription
cDNA	Complementary DNA
PCR	Polymerase Chain Reaction
TCGA	The Cancer Genome Atlas
GTEx	Genotype-Tissue Expression
GEPIA	Gene Expression Profiling Interactive Analysis
LIHC	Liver Hepatocellular Carcinoma (TCGA dataset)
EGFR	Epidermal Growth Factor Receptor
ErbB2	Erb-B2 Receptor Tyrosine Kinase 2 (also known as HER2)
BCR-Abl	Breakpoint Cluster Region-Abelson Murine Leukemia Viral Oncogene Homolog 1
PI3K/Akt	Phosphoinositide 3-Kinase/Protein Kinase B Signaling Pathway
RTKs	Receptor Tyrosine Kinases
FDA	Food and Drug Administration
NSCLC	Non-Small Cell Lung Cancer
MMP2	Matrix Metallopeptidase 2

## References

[1] Li Q., Ding C., Cao M., Yang F., Yan X., He S., Cao M., Zhang S., Teng Y., Tan N. (2024). Global Epidemiology of Liver Cancer 2022: An Emphasis on Geographic Disparities. Chin. Med. J..

[2] Bray F., Laversanne M., Sung H., Ferlay J., Siegel R.L., Soerjomataram I., Jemal A. (2024). Global Cancer Statistics 2022: GLOBOCAN Estimates of Incidence and Mortality Worldwide for 36 Cancers in 185 Countries. CA Cancer J. Clin..

[3] Sukowati C.H.C. (2019). Heterogeneity of Hepatic Cancer Stem Cells. Adv. Exp. Med. Biol..

[4] Lee J.-S., Thorgeirsson S.S. (2005). Genetic Profiling of Human Hepatocellular Carcinoma. Semin. Liver Dis..

[5] Boyault S., Rickman D.S., de Reyniès A., Balabaud C., Rebouissou S., Jeannot E., Hérault A., Saric J., Belghiti J., Franco D. (2007). Transcriptome Classification of HCC Is Related to Gene Alterations and to New Therapeutic Targets. Hepatology.

[6] Caruso S., Calatayud A.-L., Pilet J., La Bella T., Rekik S., Imbeaud S., Letouzé E., Meunier L., Bayard Q., Rohr-Udilova N. (2019). Analysis of Liver Cancer Cell Lines Identifies Agents with Likely Efficacy Against Hepatocellular Carcinoma and Markers of Response. Gastroenterology.

[7] Llovet J.M., Kelley R.K., Villanueva A., Singal A.G., Pikarsky E., Roayaie S., Lencioni R., Koike K., Zucman-Rossi J., Finn R.S. (2021). Hepatocellular Carcinoma. Nat. Rev. Dis. Prim..

[8] Llovet J.M., Ricci S., Mazzaferro V., Hilgard P., Gane E., Blanc J.-F., de Oliveira A.C., Santoro A., Raoul J.-L., Forner A. (2008). Sorafenib in Advanced Hepatocellular Carcinoma. N. Engl. J. Med..

[9] Sun M., Zhang Z., Chen C., Zhong J., Long Z., Shen L., Huang H., Lu J. (2025). Exploring the Potential Mechanisms of Sorafenib Resistance in Hepatocellular Carcinoma Cell Lines Based on RNA Sequencing. Cancer Cell Int..

[10] Cabral L.K.D., Tiribelli C., Sukowati C.H.C. (2020). Sorafenib Resistance in Hepatocellular Carcinoma: The Relevance of Genetic Heterogeneity. Cancers.

[11] Pelaz S.G., Tabernero A. (2022). Src: Coordinating Metabolism in Cancer. Oncogene.

[12] Ren H., Fang J., Ding X., Chen Q. (2016). Role and Inhibition of Src Signaling in the Progression of Liver Cancer. Open Life Sci..

[13] Cabral L.K.D., Giraudi P.J., Giannelli G., Dituri F., Negro R., Tiribelli C., Sukowati C.H.C. (2023). Network Analysis for the Discovery of Common Oncogenic Biomarkers in Liver Cancer Experimental Models. Biomedicines.

[14] Szklarczyk D., Kirsch R., Koutrouli M., Nastou K., Mehryary F., Hachilif R., Gable A.L., Fang T., Doncheva N.T., Pyysalo S. (2023). The STRING Database in 2023: Protein–Protein Association Networks and Functional Enrichment Analyses for Any Sequenced Genome of Interest. Nucleic Acids Res..

[15] Raudvere U., Kolberg L., Kuzmin I., Arak T., Adler P., Peterson H., Vilo J. (2019). G: Profiler: A Web Server for Functional Enrichment Analysis and Conversions of Gene Lists (2019 Update). Nucleic Acids Res..

[16] Weinstein J.N., Collisson E.A., Mills G.B., Shaw K.R.M., Ozenberger B.A., Ellrott K., Shmulevich I., Sander C., Stuart J.M., Cancer Genome Atlas Research Network (2013). The Cancer Genome Atlas Pan-Cancer Analysis Project. Nat. Genet..

[17] GTEx Consortium Human Genomics (2015). The Genotype-Tissue Expression (GTEx) Pilot Analysis: Multitissue Gene Regulation in Humans. Science.

[18] Tang Z., Li C., Kang B., Gao G., Li C., Zhang Z. (2017). GEPIA: A Web Server for Cancer and Normal Gene Expression Profiling and Interactive Analyses. Nucleic Acids Res..

[19] Uhlen M., Zhang C., Lee S., Sjöstedt E., Fagerberg L., Bidkhori G., Benfeitas R., Arif M., Liu Z., Edfors F. (2017). A Pathology Atlas of the Human Cancer Transcriptome. Science.

[20] Blagosklonny M.V. (2004). Analysis of FDA Approved Anticancer Drugs Reveals the Future of Cancer Therapy. Cell Cycle.

[21] Yap T.A., Omlin A., de Bono J.S. (2013). Development of Therapeutic Combinations Targeting Major Cancer Signaling Pathways. J. Clin. Oncol..

[22] Simatou A., Simatos G., Goulielmaki M., Spandidos D.A., Baliou S., Zoumpourlis V. (2020). Historical Retrospective of the SRC Oncogene and New Perspectives (Review). Mol. Clin. Oncol..

[23] Feng H. (2006). Activation of C-Yes in Hepatocellular Carcinoma: A Preliminary Study. World J. Gastroenterol..

[24] Roskoski R. (2015). Src Protein-Tyrosine Kinase Structure, Mechanism, and Small Molecule Inhibitors. Pharmacol. Res..

[25] Thomas S.M., Brugge J.S. (1997). Cellular Functions Regulated by Src Family Kinases. Annu. Rev. Cell Dev. Biol..

[26] Takahashi M., Araki T., Yashima H., Nagamine A., Nagano D., Yamamoto K. (2023). Increased c-SRC Expression Is Involved in Acquired Resistance to Lenvatinib in Hepatocellular Carcinoma. Oncol. Lett..

[27] Yao D., Deng Y., Zhang S., Liang L., Zhang L., Weng S., Chen S. (2023). Comprehensive Analysis of Prognostic Value and Immune Infiltration of Src Family Kinases in Hepatocellular Carcinoma. Front. Biosci. (Landmark Ed.).

[28] Yang J., Zhang X., Liu L., Yang X., Qian Q., Du B. (2021). C-Src Promotes the Growth and Tumorigenesis of Hepatocellular Carcinoma via the Hippo Signaling Pathway. Life Sci..

[29] Voisin L., Lapouge M., Saba-El-Leil M.K., Gombos M., Javary J., Trinh V.Q., Meloche S. (2024). Syngeneic Mouse Model of YES-Driven Metastatic and Proliferative Hepatocellular Carcinoma. Dis. Model. Mech..

[30] Steinberg M. (2007). Dasatinib: A Tyrosine Kinase Inhibitor for the Treatment of Chronic Myelogenous Leukemia and Philadelphia Chromosome-Positive Acute Lymphoblastic Leukemia. Clin. Ther..

[31] Tokarski J.S., Newitt J.A., Chang C.Y.J., Cheng J.D., Wittekind M., Kiefer S.E., Kish K., Lee F.Y.F., Borzillerri R., Lombardo L.J. (2006). The Structure of Dasatinib (BMS-354825) Bound to Activated ABL Kinase Domain Elucidates Its Inhibitory Activity against Imatinib-Resistant ABL Mutants. Cancer Res..

[32] Puls L.N., Eadens M., Messersmith W. (2011). Current Status of SRC Inhibitors in Solid Tumor Malignancies. Oncol..

[33] Hennequin L.F., Allen J., Breed J., Curwen J., Fennell M., Green T.P., Lambert-van der Brempt C., Morgentin R., Norman R.A., Olivier A. (2006). *N*-(5-Chloro-1,3-Benzodioxol-4-Yl)-7-[2-(4-Methylpiperazin-1-Yl)Ethoxy]-5-(Tetrahydro-2*H*-Pyran-4-Yloxy)Quinazolin-4-Amine, a Novel, Highly Selective, Orally Available, Dual-Specific c-Src/Abl Kinase Inhibitor. J. Med. Chem..

[34] Ramos R., Vale N. (2024). Dual Drug Repurposing: The Example of Saracatinib. Int. J. Mol. Sci..

[35] Lara P.N., Longmate J., Evans C.P., Quinn D.I., Twardowski P., Chatta G., Posadas E., Stadler W., Gandara D.R. (2009). A Phase II Trial of the Src-Kinase Inhibitor AZD0530 in Patients with Advanced Castration-Resistant Prostate Cancer: A California Cancer Consortium Study. Anticancer. Drugs.

[36] Martellucci S., Clementi L., Sabetta S., Mattei V., Botta L., Angelucci A. (2020). Src Family Kinases as Therapeutic Targets in Advanced Solid Tumors: What We Have Learned so Far. Cancers.

[37] Lombardo L.J., Lee F.Y., Chen P., Norris D., Barrish J.C., Behnia K., Castaneda S., Cornelius L.A.M., Das J., Doweyko A.M. (2004). Discovery of *N*-(2-Chloro-6-Methyl-Phenyl)-2-(6-(4-(2-Hydroxyethyl)-Piperazin-1-Yl)-2-Methylpyrimidin-4-Ylamino)Thiazole-5-Carboxamide (BMS-354825), a Dual Src/Abl Kinase Inhibitor with Potent Antitumor Activity in Preclinical Assays. J. Med. Chem..

[38] Laurie S.A., Goss G.D., Shepherd F.A., Reaume M.N., Nicholas G., Philip L., Wang L., Schwock J., Hirsh V., Oza A. (2014). A Phase II Trial of Saracatinib, an Inhibitor of Src Kinases, in Previously-Treated Advanced Non–Small-Cell Lung Cancer: The Princess Margaret Hospital Phase II Consortium. Clin. Lung Cancer.

[39] Chou T.-C. (2006). Theoretical Basis, Experimental Design, and Computerized Simulation of Synergism and Antagonism in Drug Combination Studies. Pharmacol. Rev..

[40] Dor I., Namba M., Sato J. (1975). Establishment and Some Biological Characteristics of Human Hepatoma Cell Lines. Gan.

[41] Arii S., Mise M., Harada T., Furutani M., Ishigami S., Niwano M., Mizumoto M., Fukumoto M., Imamura M. (1996). Overexpression of Matrix Metalloproteinase 9 Gene in Hepatocellular Carcinoma with Invasive Potential. Hepatology.

[42] Pinto E., Pelizzaro F., Cardin R., Battistel M., Palano G., Bertellini F., Kitenge M.P., Peserico G., Farinati F., Russo F.P. (2024). HIF-1α and VEGF as Prognostic Biomarkers in Hepatocellular Carcinoma Patients Treated with Transarterial Chemoembolization. Dig. Liver Dis..

[43] Huang S., Sinicrope F.A. (2010). Sorafenib Inhibits STAT3 Activation to Enhance TRAIL-Mediated Apoptosis in Human Pancreatic Cancer Cells. Mol. Cancer Ther..

[44] Kim L.C., Song L., Haura E.B. (2009). Src Kinases as Therapeutic Targets for Cancer. Nat. Rev. Clin. Oncol..

[45] Yu H., Pardoll D., Jove R. (2009). STATs in Cancer Inflammation and Immunity: A Leading Role for STAT3. Nat. Rev. Cancer.

[46] Abu-Ghazaleh R., Kabir J., Jia H., Lobo M., Zachary I. (2001). Src Mediates Stimulation by Vascular Endothelial Growth Factor of the Phosphorylation of Focal Adhesion Kinase at Tyrosine 861, and Migration and Anti-Apoptosis in Endothelial Cells. Biochem. J..

[47] Eliceiri B.P., Paul R., Schwartzberg P.L., Hood J.D., Leng J., Cheresh D.A. (1999). Selective Requirement for Src Kinases during VEGF-Induced Angiogenesis and Vascular Permeability. Mol. Cell.

[48] Mitra S.K., Hanson D.A., Schlaepfer D.D. (2005). Focal Adhesion Kinase: In Command and Control of Cell Motility. Nat. Rev. Mol. Cell Biol..

[49] Jeong K.-Y., Park M., Sim J.-J., Kim H.M. (2020). Combination Antitumor Effect of Sorafenib via Calcium-Dependent Deactivation of Focal Adhesion Kinase Targeting Colorectal Cancer Cells. Molecules.

[50] Zhou Q., Guo X., Choksi R. (2017). Activation of Focal Adhesion Kinase and Src Mediates Acquired Sorafenib Resistance in A549 Human Lung Adenocarcinoma Xenografts. J. Pharmacol. Exp. Ther..

[51] Bustin S.A., Benes V., Garson J.A., Hellemans J., Huggett J., Kubista M., Mueller R., Nolan T., Pfaffl M.W., Shipley G.L. (2009). The MIQE Guidelines: Minimum Information for Publication of Quantitative Real-Time PCR Experiments. Clin. Chem..

[52] Cancer Genome Atlas Research Network (2017). Cancer Genome Atlas Research Network Comprehensive and Integrative Genomic Characterization of Hepatocellular Carcinoma. Cell.

