# Supplementary material for: Dual Inhibition of SRC Family Kinases and Sorafenib Enhances Anti-Tumor Activity in Hepatocellular Carcinoma Cells
Source: Int J Mol Sci. 2025 Jul 6;26(13):6506. doi: 10.3390/ijms26136506 (PMC12250370; doi:10.3390/ijms26136506)
Supplement: Supplementary file 1 [file ijms-26-06506-s001.zip › ijms-3702895-supplementary.pdf]

SUPPLEMENTAL DATA

SRC Family Kinase Inhibition Potentiates Sorafenib Efficacy in Hepatocellular Carcinoma

Loraine Kay Cabral[1,2], Cyrollah Disoma[1,3], Paola Tarchi[4], Korri Elvanita El-Khobar[5], Agustiningsih Agustiningsih[5], Francesco Dituri[6], Claudio Tiribelli[1], Caecilia Sukowati[1,5,*]

1 Liver Cancer Unit, Fondazione Italiana Fegato ONLUS, AREA Science Park, Campus Basovizza, 34149 Trieste, Italy; cyrollah.disoma@fegato.it; ctliver@fegato.it; caecilia.sukowati@fegato.it

2 Philippine Council for Health Research and Development, Department of Science and Technology, Saliksik Building, Science Community Complex General Santos Ave., Bicutan, 1631 Taguig City, Philippines; ldcabral@pchrd.dost.gov.ph

3 Doctoral School of Molecular Biomedicine, Department of Life Sciences, University of Trieste, 34149 Trieste, Italy

4 General Surgery Department, Azienda sanitaria universitaria Giuliano Isontina, 34128 Trieste, Italy; paola.tarchi@asugi.sanita.fvg.it

5 Eijkman Research Center for Molecular Biology, Research Organization for Health, National Research and Innovation Agency of Indonesia (BRIN), Jakarta Pusat 10340, Indonesia; korr001@brin.go.id; agustiningsih@brin.go.id

6 IRCCS National Institute of Gastroenterology "S. De Bellis" Research Hospital Via Turi 27 - 70013 Bari, Italy; francesco.dituri@irccsdebellis.it

* Correspondence: caecilia.sukowati@fegato.it; Tel.: +39-040-3757926

Academic Editor: Firstname Lastname

Received: date
Revised: date
Accepted: date
Published: date

Citation: To be added by editorial staff during production.

Copyright: © 2025 by the authors. Submitted for possible open access publication under the terms and conditions of the Creative Commons Attribution (CC BY) license (https://creativecommons.org/licenses/by/4.0/).

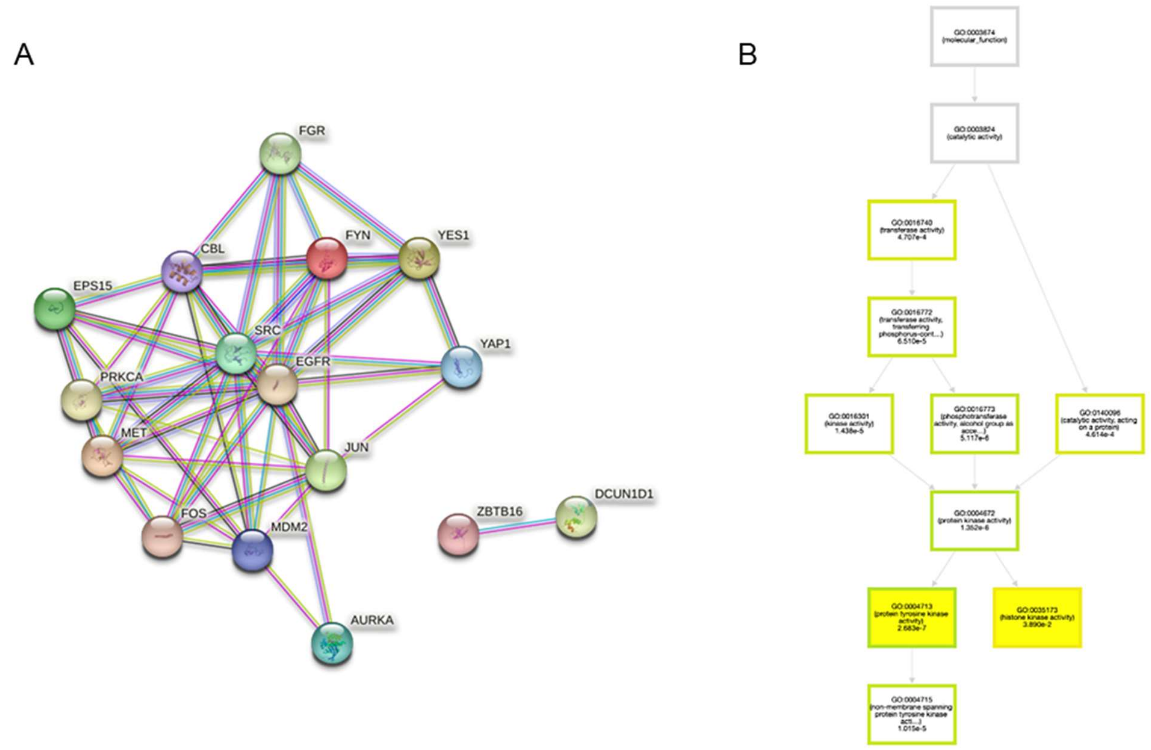

Figure S1. Bioinformatics analysis for the selection of SFKs as targets [1]. (**a**) Enrichment network interaction of the 16 proto-oncogene targets using STRING database [2]. (**b**) Gene enrichment analysis using g.profiler [3].

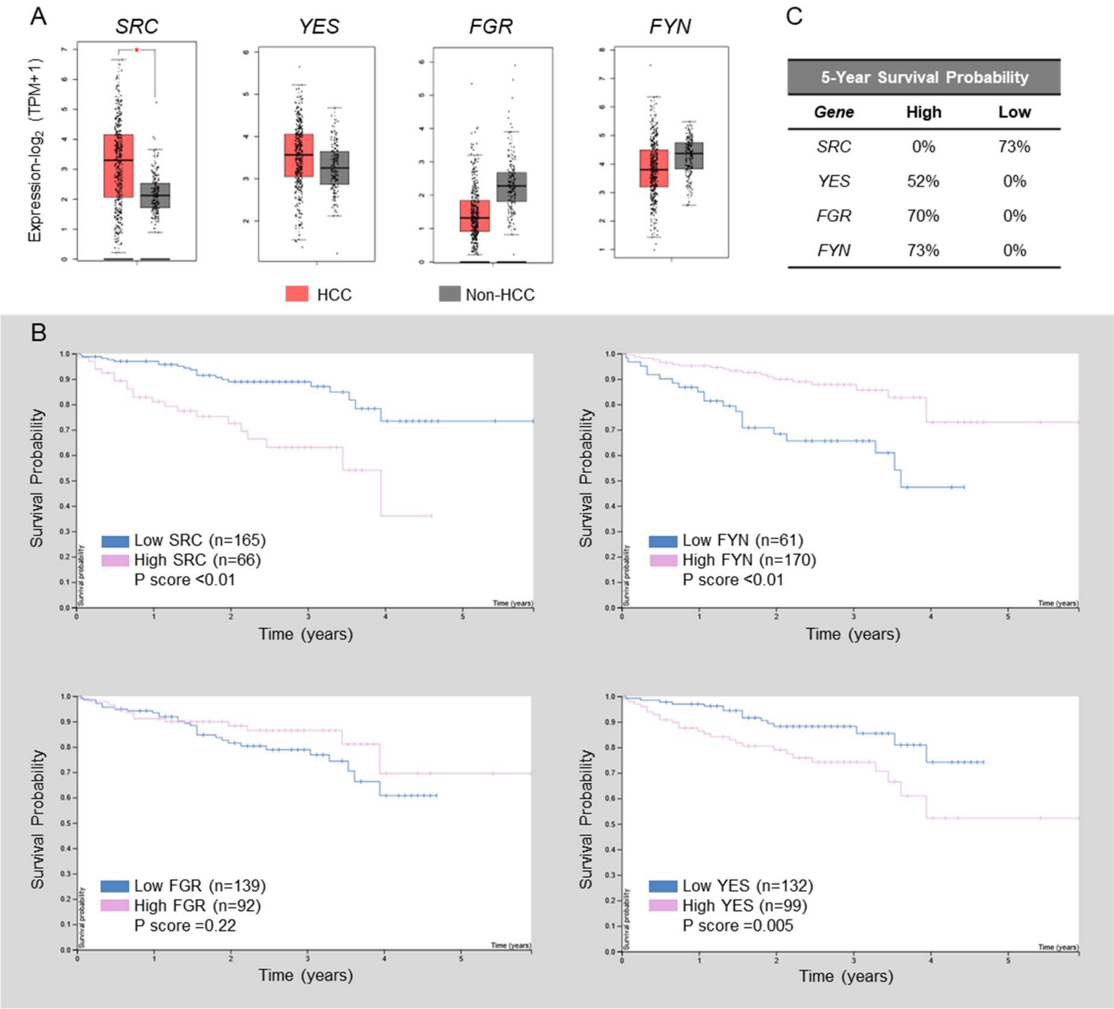

5-Year Survival Probability		
Gene	High	Low
SRC	0%	73%
YES	52%	0%
FGR	70%	0%
FYN	73%	0%

Figure S2. Gene expressions of SFKs members *SRC, YES1, FGR*, and *FYN* in TCGA Liver Hepatocellular Carcinoma (TCGA LIHC) and Genotype Tissue Expression (GTEx) cohorts. (**a**) Normalized expressions of SFKs genes in tumor tissues (n=369) vs normal liver tissues (n=160) from TCGA and GTEx datasets, accessed through GEPIA2 database (**b**) Survival probability obtained from The Human Protein Atlas. (**c**) 5-year survival probability. Data were retrieved from [4–7].

References

1. Cabral, L.K.D.; Giraudi, P.J.; Giannelli, G.; Dituri, F.; Negro, R.; Tiribelli, C.; Sukowati, C.H.C. Network Analysis for the Discovery of Common Oncogenic Biomarkers in Liver Cancer Experimental Models. *Biomedicines* **2023**, *11*, 342, doi:10.3390/biomedicines11020342.
2. Szklarczyk, D.; Kirsch, R.; Koutrouli, M.; Nastou, K.; Mehryary, F.; Hachilif, R.; Gable, A.L.; Fang, T.; Doncheva, N.T.; Pyysalo, S.; et al. The STRING Database in 2023: Protein–Protein Association Networks and Functional Enrichment Analyses for Any Sequenced Genome of Interest. *Nucleic Acids Res.* **2023**, *51*, D638–D646, doi:10.1093/nar/gkac1000.
3. Raudvere, U.; Kolberg, L.; Kuzmin, I.; Arak, T.; Adler, P.; Peterson, H.; Vilo, J. G:Profiler: A Web Server for Functional Enrichment Analysis and Conversions of Gene Lists (2019 Update). *Nucleic Acids Res.* **2019**, *47*, W191–W198, doi:10.1093/nar/gkz369.
4. Cancer Genome Atlas Research Network. Electronic address: wheeler@bcm.edu; Cancer Genome Atlas Research Network Comprehensive and Integrative Genomic Characterization of Hepatocellular Carcinoma. *Cell* **2017**, *169*, 1327-1341.e23, doi:10.1016/j.cell.2017.05.046.
5. GTEx Consortium Human Genomics. The Genotype-Tissue Expression (GTEx) Pilot Analysis: Multitissue Gene Regulation in Humans. *Science* **2015**, *348*, 648–660, doi:10.1126/science.1262110.
6. Tang, Z.; Li, C.; Kang, B.; Gao, G.; Li, C.; Zhang, Z. GEPIA: A Web Server for Cancer and Normal Gene Expression Profiling and Interactive Analyses. *Nucleic Acids Res.* **2017**, *45*, W98–W102, doi:10.1093/nar/gkx247.
7. Uhlen, M.; Zhang, C.; Lee, S.; Sjöstedt, E.; Fagerberg, L.; Bidkhori, G.; Benfeitas, R.; Arif, M.; Liu, Z.; Edfors, F.; et al. A Pathology Atlas of the Human Cancer Transcriptome. *Science* **2017**, *357*, eaan2507, doi:10.1126/science.aan2507.
